# Who is in the driver's seat? *Parvimonas micra* : An understudied pathobiont at the crossroads of dysbiotic disease and cancer

**DOI:** 10.1111/1758-2229.13153

**Published:** 2023-03-30

**Authors:** Dustin L. Higashi, Madeline C. Krieger, Hua Qin, Zhengzhong Zou, Elizabeth A. Palmer, Jens Kreth, Justin Merritt

**Affiliations:** ^1^ Department of Restorative Dentistry Oregon Health and Science University Portland Oregon USA; ^2^ Department of Pediatric Dentistry Oregon Health and Science University Portland Oregon USA; ^3^ Department of Molecular Microbiology and Immunology Oregon Health and Science University Portland Oregon USA

## Abstract

Recent advances in our understanding of microbiome composition at sites of inflammatory dysbiosis have triggered a substantial interest in a variety of historically understudied bacteria, especially among fastidious obligate anaerobes. A plethora of new evidence suggests that these microbes play outsized roles in establishing synergistic polymicrobial infections at many different sites in the human body. *Parvimonas micra* is a prime example of such an organism. Despite being almost completely uncharacterized at the genetic level, it is one of the few species commonly detected in abundance at multiple mucosal sites experiencing either chronic or acute inflammatory diseases, and more recently, it has been proposed as a discriminating biomarker for multiple types of malignancies. In the absence of disease, *P. micra* is commonly found in low abundance, typically residing within the oral cavity and gastrointestinal tract. *P. micra* exhibits the typical features of an inflammophilic organism, meaning its growth actually benefits from active inflammation and inflammatory tissue destruction. In this mini‐review, we will describe our current understanding of this underappreciated but ubiquitous pathobiont, specifically focusing upon the role of *P. micra* in polymicrobial inflammatory dysbiosis and cancer as well as the key emerging questions regarding its pathobiology. Through this timely work, we highlight *Parvimonas micra* as a significant driver of disease and discuss its unique position at the crossroads of dysbiosis and cancer.

## INTRODUCTION

Recent advances in next‐generation sequencing (NGS) technologies have triggered an explosion of culture‐independent analyses of the microbial communities found at numerous sites of both health and disease around the human body. Such studies have rapidly reshaped our perception of the microbial ecology found at these locations, especially among fastidious, slow growing, and/or uncultivated organisms, many of which are newly discovered and completely uncharacterized. Importantly, recent microbiome studies have revealed a surprising number of diseases that correlate with the presence of dysbiotic microbiota. For example, dysbiosis has been linked to diverse conditions such as periodontal disease, diabetes, obesity, hair loss, irritable bowel syndrome, chronic lung infections, urogenital tract infections, various cancers, and so on (Abusleme et al., 2013; Bielka et al., 2022; Blohs et al., 2023; Chong et al., 2019; Helmink et al., 2019; Mestrovic et al., 2020; Sakamoto et al., 2021; Yu et al., 2022). Studies targeting the pathogenic mechanisms triggered by dysbiosis represent the next frontier in microbiome research. Before such a transition can occur, the field must first identify the key drivers of pathogenesis within these communities and then develop new genetic tools to study them, as only a minute fraction of the human microbiome has ever been interrogated at the genetic level.

*Parvimonas micra* is a prominent example of such an organism, as it remains largely uncharacterized, despite an ever‐increasing number of microbiome studies linking it to a wide variety of dysbiotic diseases. *P. micra* is a fastidious Gram‐positive obligate anaerobe and is a common constituent of multiple mucosal sites in the human body, primarily within the oral cavity and to a lesser extent, the gastrointestinal tract (Finegold et al., 1974; Rams et al., 1992). It is among the most highly enriched species in numerous studies of mucosal inflammatory disease (Murphy & Frick, 2013). It is also commonly one of the most abundant species found in systemic abscesses and exhibits a strong association with a variety of malignant tumours (Coker et al., 2018; Flemer et al., 2018; Saffarian et al., 2019). In the oral cavity, *P. micra* exists as a ubiquitous low abundance species during oral health, but it is highly enriched in periodontitis lesions, infected root canals, and is particularly prevalent in odontogenic (i.e. oral) abscesses (Colombo et al., 2009; Gomes et al., 2015; Nonnenmacher et al., 2004; Turng et al., 1996). This mini‐review will focus on the role of *P. micra* in oral dysbiotic disease, examine its recent association with cancer, and discuss critical outstanding questions for future *P. micra* genetic research.

### P. micra *ecology and taxonomy*

**FIGURE 1:**
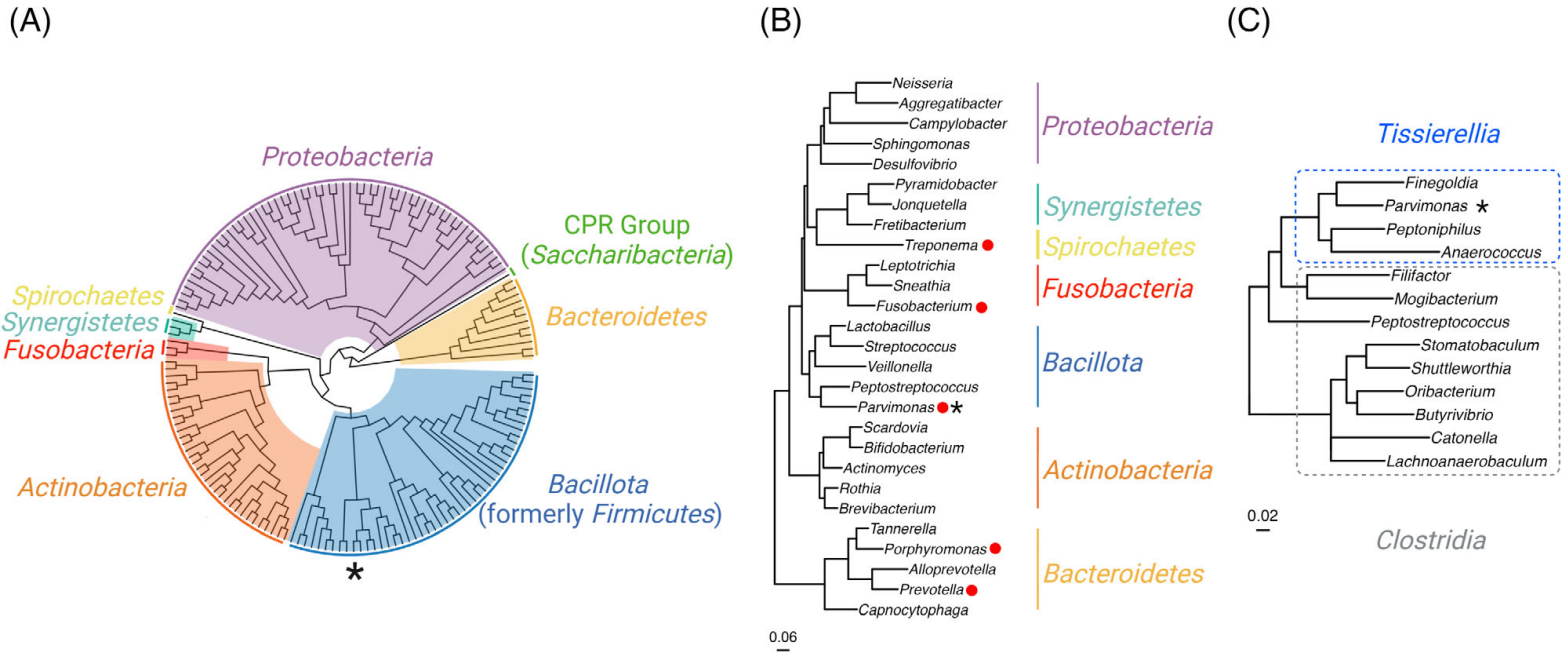
Taxonomy of *Parvimonas micra*. (A) Global phylogeny of organisms from the expanded Human Oral Microbiome Database (http://www.ehomd.org). (B) Phylogenetic tree illustrating a number of genera (red dots) commonly associated with dental disease. (C) Phylogenetic tree illustrating the reclassification of *P. micra* out of the *Clostridia* class (grey dashed line) and into *Tissierellia* (blue dashed line). “*” indicates *P. micra*. Maximum‐likelihood phylogenetic trees were computed in RAxML using the GTR GAMMA substitution model and rapid bootstrapping with 100 replicates using genomes curated from the HOMD 16S rDNA sequence database.

*P. micra* is a member of the mostly uncharacterized *Tissierellia* class within the *Bacillota* (formerly *Firmicutes*) phylum. *P. micra* is also included among the Gram‐positive anaerobic cocci (GPAC) group, which accounts for the majority of anaerobic bacteria isolated from clinical specimens (Murphy & Frick, 2013). For many years, *P. micra* was classified as a member of the *Peptostreptococcus* genus (referred to as *Peptostreptococcus micros*) and then was later renamed as *Micromonas micros*, placing it within the *Clostridia* class. However, differences in both genotypic and phenotypic characteristics have resulted in its taxonomic reclassification within the *Tissierellia* class as well as the creation of a unique genus, *Parvimonas*, for which *P. micra* was the first named species (Murdoch & Shah, 1999; Tindall & Euzeby, 2006) (Figure 1A–C). *P. micra* can be found during episodes of inflammation in the oral cavity, gastrointestinal tract, respiratory system, and/or urogenital tract (Brook, 1988; Rams et al., 1992; The et al., 2019). It is also often identified in infections located at other sites like the brain, spine, liver, and blood (Brook, 1988; Murdoch et al., 1988). Even though it is now recognized that *P. micra* exists at many different sites of mucosal infections and other diseases, the vast majority of *P. micra* pathogenesis studies have focused upon its role in oral infections.

### P. micra *in chronic and acute oral disease*

The microbial diversity in the human oral microbiome is only surpassed by that of the gut (Pasolli et al., 2019; Tierney et al., 2019). The oral microbiome is also one of the best characterized. In fact, the critical connection between oral health and oral microbial ecology was recognized more than a century ago (He & Shi, 2009; Merritt & Kreth, 2022), largely aided by the relative ease of clinical sampling in the oral cavity and the extreme prevalence of dysbiotic oral diseases like caries (tooth decay) and periodontitis (chronic inflammation of the gingiva). Here, we will discuss the current knowledge of *P. micra* in the context of two oral diseases, periodontitis and odontogenic abscesses.

**FIGURE 2:**
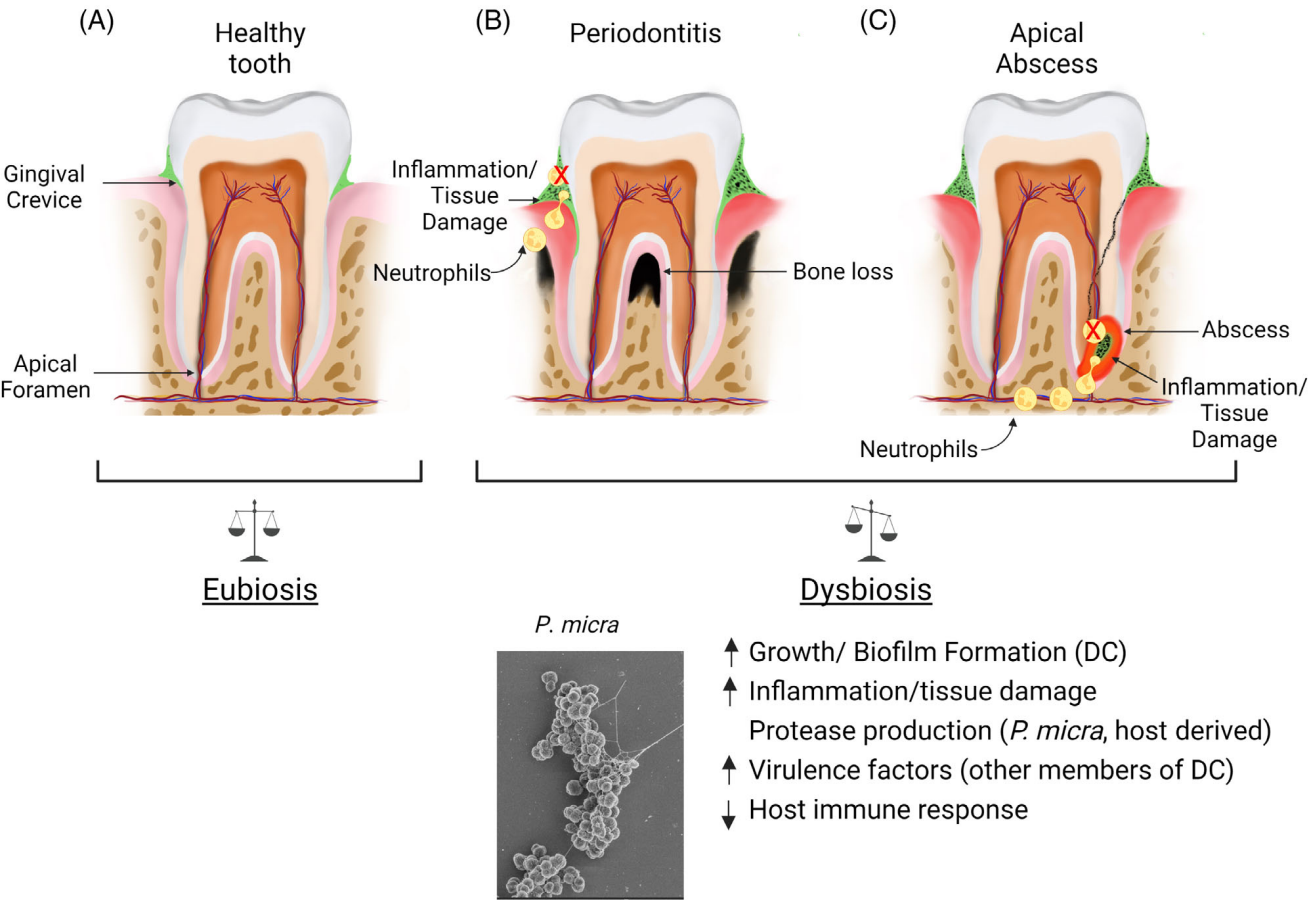
Enrichment of *Parvimonas micra* in oral disease. (Top) Shifts in the (A) health‐associated eubiotic community can lead to (B) periodontitis and (C) apical abscess formation. Alterations to the composition of the healthy microbiome of the gingival crevice (green) results in the inflammatory enrichment of *P. micra* and other members of the dysbiotic community (black). (Bottom) *P. micra*‐induced impacts upon the dysbiotic community (DC) and host environment.

Periodontitis is one of the most prevalent chronic infectious diseases of humanity, affecting >500 million people worldwide (Kassebaum et al., 2017). During oral health, the microbiome promotes homeostasis by limiting acidification of the oral biofilm and by preventing destructive inflammation of the gingiva (Sedghi et al., 2021) (Figure 2A). In contrast, periodontitis is characterized by excessive chronic gingival inflammation leading to the subsequent resorption of the alveolar bone and destruction of the connective tissue supporting the tooth (Figure 2B). Multiple microbiome studies have revealed an enrichment of core organisms associated with oral health and periodontitis. While variability exists between these studies, certain recurring groups are commonly found associated with healthy microbiomes, such as certain *Actinomyces* spp., *Rothia* spp., and *Streptococcus sanguinis*, whereas periodontitis‐associated microbiomes typically contain an overrepresentation of organisms such as *Treponema denticola*, *Porphyromonas gingivalis*, *Prevotella intermedia*, *Fusobacterium nucleatum*, *Parvimonas micra*, and others (Abusleme et al., 2013; Boutin et al., 2017; Kirst et al., 2015; Lourenco et al., 2014).

*P. micra* is also frequently observed within multispecies communities associated with acute odontogenic infections such as periapical abscesses (Figure 2C). Periapical abscesses are caused by an infection of the dental pulp within the root canal of the tooth and are the most common type of odontogenic abscess (Siqueira & Rocas, 2013, 2022). Tooth abscesses are also one of the most common sources of nontraumatic dental emergencies (Quinonez et al., 2009). In these infections, NGS and culture‐based methods have identified many of the same organisms found in periodontitis (e.g. *Parvimonas* sp., *Fusobacterium* spp., *Prevotella* spp., *Treponema* spp., *Porphyromonas* spp., etc.). While many of these microbes may be considered significant pathobionts, metagenomic studies underscore the fact that most chronic and acute oral diseases are the result of complex synergistic polymicrobial communities, rather than due to the singular action of any particular organism (Siqueira & Rocas, 2022). Even so, it is clear that *P. micra* benefits tremendously from the inflammatory growth environments found in both periodontitis and odontogenic abscesses. It is worth noting that in either chronic or acute oral disease, an increased prevalence of *P. micra* is correlated with both the presence of disease and its severity as well (Abusleme et al., 2013; Rocas & Siqueira, 2018; Santos et al., 2011). This suggests that *P. micra* may not only influence the establishment of the initial infection but may also help to drive the progression of inflammatory diseases. How might this occur? Our present mechanistic understanding of these associations unfortunately remains fairly rudimentary, but the field has revealed a variety of intriguing aspects of *P. micra* pathobiology that will likely become major topics of future genetic research.

### *The role of* P. micra *in polymicrobial oral biofilms*

Members of mucosal polymicrobial communities often display remarkable levels of synergism that enhance their colonization, persistence, and/or pathogenicity (Lamont et al., 2018). Furthermore, physical interactions in these communities can facilitate crucial metabolic exchanges and provide protection from both host‐derived and environmental insults. The ability for certain species to colonize various niches within the host provides clues to the mechanisms used to establish these communities. Accordingly, a number of studies have hinted at a key role for *P. micra* in the development of dysbiotic infections.

Cell scrapings of subgingival crevicular cells have demonstrated the direct physical association of *P. micra* with oral epithelial cells in vivo. This was further confirmed with in vitro adhesion assays using HeLa cells and primary gingival epithelial cells (Dibart et al., 1998; Dzink et al., 1989). *P. micra*–host cell interactions were shown to be mediated by extracellular polysaccharides located on the bacterial cell surface (Kremer et al., 1999). The ability of *P. micra* to adhere to epithelial cells may facilitate its spread to new sites in the oral mucosa, and perhaps even seed the development of polymicrobial communities through interspecies coaggregation/coadhesion interactions. Distinct patterns have been described for the spatial distribution of the multi‐genus consortia found within supragingival dental plaque specimens (Mark Welch et al., 2016), while numerous coaggregation studies have provided the molecular basis for modelling the accretion of these biofilm communities (Jakubovics, 2015; Kolenbrander & London, 1993; Valm, 2019). *P. micra* was shown to coaggregate with *T. denticola* and can serve as a substrate for *T. denticola* adherence (Cogoni et al., 2012). *P. micra* can also coaggregate with *P. gingivalis* and *F. nucleatum* (Horiuchi et al., 2020; Kremer & van Steenbergen, 2000) as well as utilize soluble factors from both organisms to enhance its own growth during coculture. Conversely, *P. micra* also releases soluble factors that enhance the biofilm formation of *P. gingivalis* and *F. nucleatum* (Horiuchi et al., 2020). A dual‐species coculture model demonstrated how *P. micra* is specifically able to stimulate the growth of *P. gingivalis*, unlike cocultures containing *F. nucleatum* and *Streptococcus oralis* (Neilands et al., 2019). It was postulated that the observed growth enhancement was due to the secretion of glycolytic enzymes and other proteins involved in amino acid and butyric acid metabolism. In addition to nutrient exchange, polymicrobial *P. micra*‐containing biofilms can display an enhanced resistance to environmental insults as well. In mixed biofilms of *P. micra* and *F. nucleatum*, both organisms displayed a decreased sensitivity to sodium hypochlorite treatments as compared to their respective single species biofilms. This resistance increased with the age of the biofilm, suggesting a time‐dependent synergy between the organisms (Ozok et al., 2007). It is conceivable that such synergistic oxidative stress resistance could similarly provide *P. micra*‐containing polymicrobial biofilms with enhanced resistance to oxidative host defences like those produced by neutrophil granulocytes. *P. micra* can also influence the production of virulence factors from other coinfecting organisms. For example, *P. micra* is a potent stimulator of both *P. gingivalis* growth and its production of gingipains, which are secreted proteases that have a myriad of pathogenesis functions, including adhesion to host cells, the utilization of nutrients, the development of biofilms, and the disruption of host immune signalling due to their cleavage of proinflammatory cytokines like IL‐2, IL‐1β, TNF‐α, IL‐6, and IL‐8 (Chow et al., 2022; Khalaf & Bengtsson, 2012). In a recent in vitro study, the expression of gingipain activity was determined using an 8‐species consortium consisting of *P. gingivalis*, *F. nucleatum*, *Actinomyces naeslundii*, *Streptococcus oralis*, *Streptococcus gordonii*, *Streptococcus mitis*, *Streptococcus cristatus*, and *P. micra*. Despite the numerous organisms present in the model, gingipain activity was specifically enhanced by the presence of *P. micra*. In fact, dual species *P. gingivalis‐P. micra* cocultures yielded >35‐fold increased gingipain activity compared to *P. gingivalis* alone (Neilands et al., 2019). The ability of *P. micra* to specifically induce the expression of a key virulence factor in another organism within a complex community perfectly exemplifies the polymicrobial synergism that characterizes many dysbiotic diseases.

In vivo studies have similarly demonstrated synergistic pathogenesis within cocultures containing *P. micra* (Araki et al., 2004; Sundqvist et al., 1979; van Dalen et al., 1998). Subcutaneous murine coinfections with *P*. *micra* and *P. intermedia* resulted in more virulent experimental abscesses as compared to the corresponding single species infections. Interestingly, there was no evidence of growth stimulation within the in vitro cocultures, suggesting the increased abundance of *P. intermedia* in the mixed infection abscesses was due to *P. micra* specifically providing a selective growth/survival advantage within the host (van Dalen et al., 1998). Furthermore, the abscess pus derived from the mixed infections was suitable for establishing abscesses in naive animals, whereas material derived from the individual infections was not. In another polymicrobial infection study using guinea pigs, *P. micra* was also critical for establishing abscesses in complex mixtures of oral bacteria containing up to nine different species, including *F. nucleatum* and *Prevotella melaninogenica* (Sundqvist et al., 1979). In both of these aforementioned studies, the transmissibility of *P. micra* and partner microbes contained within the experimental abscess material illustrates both the virulence and stability of the abscess communities containing *P. micra*.

### P. micra *and the host response to oral infections*

Inflammophilic bacteria are largely resistant to clearance via innate immune mechanisms and actually benefit from the presence of inflammation due to the nutrients provided as a consequence of secondary host tissue destruction (Bartold & Van Dyke, 2017; Hajishengallis, 2015). Consequently, inflammation can effectively remodel microbial communities to favour the presence of inflammophilic organisms like those often found in periodontitis lesions and odontogenic abscesses. In many cases, inflammophilic bacteria also directly stimulate innate immune mechanisms, which is presumably a key step leading to the dysbiotic remodelling of microbiome communities (Lamont et al., 2018). *P. micra* produces a number of different inflammatory mediators. For example, *P. micra* genomic DNA serves as a TLR‐9 agonist in human gingival fibroblasts, resulting in the production of TNFα and IL‐6 (Nonnenmacher et al., 2003). The *P. micra* cell wall elicits a proinflammatory response in human macrophages, inducing the secretion of TNFα, IL‐8, IL‐6, IL‐1β, and RANTES (Tanabe et al., 2007). Cell wall extracts also induce human macrophages to secrete matrix metalloproteinase 9 (MMP‐9), which plays well‐characterized roles in inflammatory tissue damage (Hannas et al., 2007).

In a polymicrobial community setting, *P. micra* may further assist other members of a dysbiotic community to survive innate immunity. In a murine infection model, *P. micra* was shown to evade neutrophil killing by dampening the production of reactive oxygen species (ROS) and impairing phagocytosis activity. Interestingly, this ability was further enhanced by coculture with *F. nucleatum*, *P. intermedia*, and *Streptococcus intermedius* (Matsui et al., 2014). Furthermore, the ability of *P*. *micra* to bind the Fc fragment of IgG may deplete the local pool of opsonins to suppress phagocytosis of other bacteria within the community (Grenier & Michaud, 1994).

The capacity of different organisms to utilize human serum components for growth may represent another key phenotype for the development of dysbiotic communities, particularly those that exhibit high proteolytic activity. During periodontitis, the expansion of the subgingival crevice into a bona fide periodontal pocket results in the increased local availability of gingival crevicular fluid (GCF), a type of serum exudate that nourishes subgingival microbiome communities (Fatima et al., 2021). In acute inflammatory conditions like the periapical abscess, invading microbes are exposed to components of the blood as a consequence of pulpitis‐derived tissue destruction within infected root canals (Galler et al., 2021). The inherent ability of *P. micra* to effectively metabolize serum components was previously illustrated in a growth enrichment study in which subgingival plaque specimens were passaged through serum. Following passaging, the resulting mixture of organisms was dominated by *P. micra* and to a lesser extent by *Prevotella* spp. and *F. nucleatum*. Furthermore, this consortium was able to proteolytically degrade IgG, IgA, and IgM as well as the complement proteins C3c and C4 (ter Steeg et al., 1987). *P. micra* proteolysis of other eukaryotic substrates was demonstrated in a study comparing two morphotypes of *P. micra*, a rough fimbriated strain and a smooth non‐fimbriated version. The rough phenotype exhibited both chymotrypsin‐like and gelatinase activities that were attributed to three distinct proteases (Grenier & Bouclin, 2006). Furthermore, the production of these proteases allowed *P. micra* to more effectively penetrate a reconstituted basement membrane. It is also worth noting that *P. micra* was found to exhibit the strongest and most diverse proteolytic activity among an extensive collection of GPAC organisms (Murdoch & Mitchelmore, 1991). In addition to producing proteases, *P. micra* may also usurp endogenous host proteases. Plasminogen is ubiquitous in human blood and is the inactive precursor to plasmin, an enzyme responsible for the breakdown of fibrin clots and other blood plasma proteins (Keragala & Medcalf, 2021). Both morphotypes of *P. micra* were shown to bind human plasminogen (Grenier & Bouclin, 2006). Treatment of plasminogen‐coated *P. micra* with either bacterial streptokinase or host‐derived urokinase resulted in surface‐associated plasmin activity that further enhanced *P. micra* penetration of reconstituted basement membranes.

### P. micra *in cancer*

Cancer is one of the most widely recognized global public health challenges, with over 19 million new cases worldwide and nearly 10 million deaths in the year 2020 alone (Sung et al., 2021). Microbial associations with certain types of cancer have been noted for a number of decades (Parsonnet et al., 1991). However, recent cancer microbiome studies have revealed that a surprising number of tumours harbour infecting bacteria, while the list of cancer‐associated microbes has been steadily growing as well (van Elsland & Neefjes, 2018). Metagenomic studies have revealed major ecological shifts in the microbiomes of healthy versus cancerous tissues, which has spurred major interest in the mechanisms by which dysbiotic communities influence tumour biology.

Recent studies have independently identified *P. micra* as a discriminating biomarker for various types of gastric, oral, and colorectal cancers (CRC) (Coker et al., 2018; Galeano Nino et al., 2022; Yang et al., 2018; Yao et al., 2021). Of these, CRC is the most frequently diagnosed and lethal (Sung et al., 2021). Metagenomic studies of CRC have found *P. micra* to be significantly enriched in both faecal samples and tissue biopsies of patients (Purcell et al., 2017; Senthakumaran et al., 2023; Yachida et al., 2019). It has been suggested that *P. micra* exhibits the strongest correlation with consensus molecular subtype 1 CRC (Purcell et al., 2017). Furthermore, the abundance of *P. micra* was identified as a significant predictor of poor treatment outcomes in CRC patients (Zhao et al., 2022). Likewise, *P. micra* abundance within hypopharyngeal squamous cell carcinomas was similarly found to correlate with tumour severity (Lau et al., 2022). Interestingly, tumour microbiomes are typically polymicrobial and appear to share many of the same overrepresented inflammophilic oral bacteria commonly associated with the dysbiotic microbiomes in periodontitis and tooth abscesses. For instance, CRC studies of large patient cohorts having diverse ethnic and geographic representation confirmed the enrichment of *P. micra* in CRC tumours but also identified a number of other inflammophilic oral bacteria such as *F. nucleatum*, *P. intermedia*, and others (Dai et al., 2018; Yu et al., 2017). Similar results were also noted in a recent microbiome analysis of six independent CRC cohorts, where *P. micra*, *F. nucleatum*, and several other oral bacteria were among the small number of species that were overrepresented in multiple CRC cohorts (Zhao et al., 2021). Perhaps less surprisingly, these same trends have been reported for oesophageal squamous cell carcinomas as well (Nomburg et al., 2022). It remains to be determined why such an overlap exists between the oral pathobionts found in multiple dysbiotic diseases and various tumours, but it is certainly conceivable that analogous mechanisms of polymicrobial synergism could be a factor.

**FIGURE 3:**
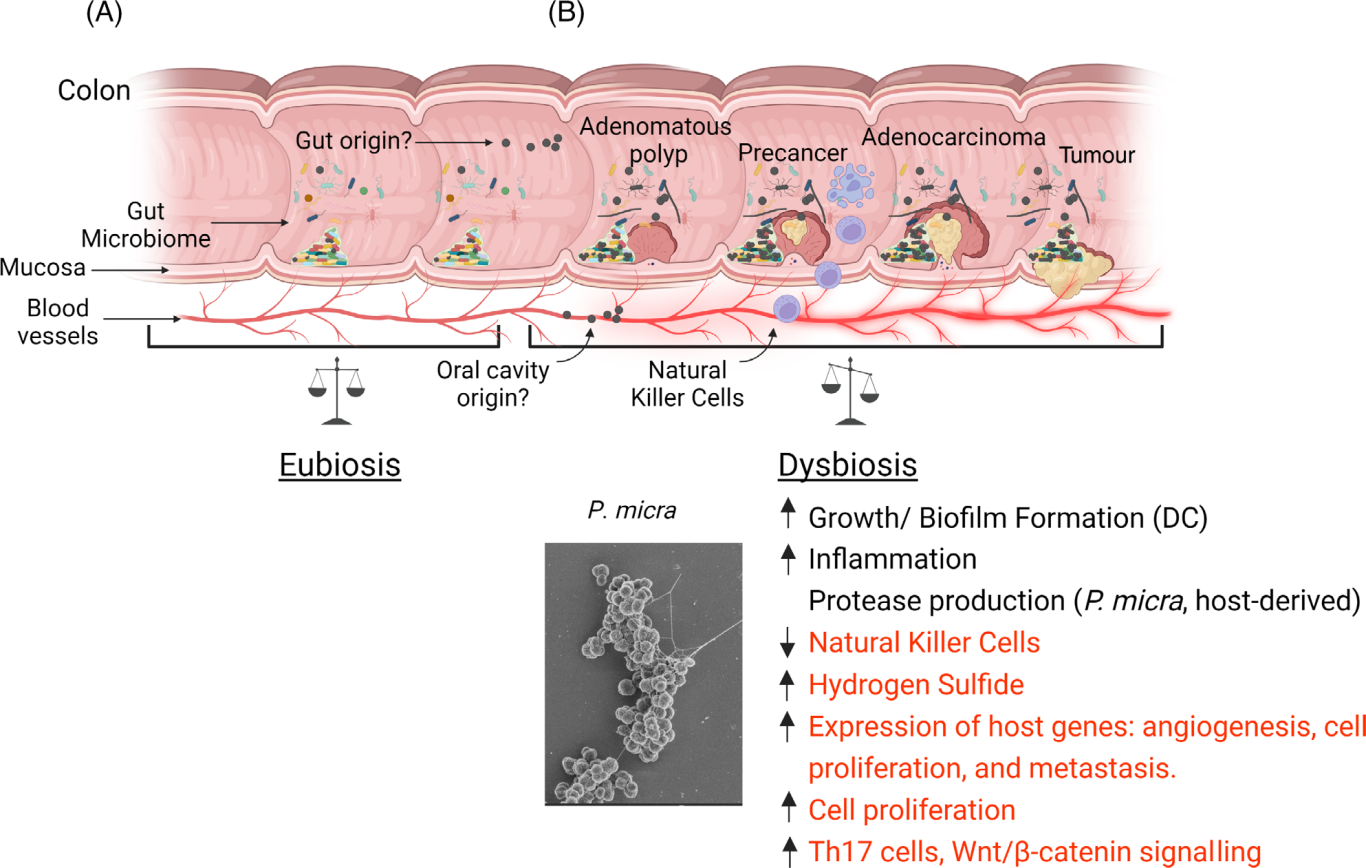
Enrichment of *Parvimonas micra* in cancer. (Top) (A) Health‐associated eubiotic community of the gut and (B) intestinal dysbiosis corresponding to an increase in a number of oral pathobionts and the development of colorectal cancer (CRC). As colorectal polyps progress from mild to severe dysplasia, there is an associated local increase in the dysbiotic alteration of gut microbiome composition, resulting in an enrichment of various inflammophilic oral bacteria, such as *P. micra* (black). (Bottom) *P. micra*‐induced influences on the dysbiotic community (DC) and host environment of CRC (red font, additional to those described in oral infections).

*P. micra* is likely to influence the development of cancer through a variety of mechanisms that still await further elucidation (Figure 3). Despite our limited mechanistic understanding of this process, some interesting themes are already emerging. First, as with various other dysbiotic diseases, inflammation can also play major roles in tumour development (Chang et al., 2023; Grivennikov et al., 2010). As mentioned earlier, *P. micra* can induce the expression of proinflammatory cytokines in multiple cell types (Nonnenmacher et al., 2003; Tanabe et al., 2007). *P. micra* was also shown to exhibit cytotoxicity towards NK cells (Kim et al., 2021), which are key immune cells involved in suppressing tumour development (Wolf et al., 2022). In a transgenic mouse model (*Apc* ^min/+^) of CRC, the introduction of *P. micra* via gavage resulted in a significant increase in both tumour prevalence and size as compared to *E. coli* and broth controls (Zhao et al., 2022). In conventional C57BL/6 mice, the introduction of *P. micra* was associated with increased colonic epithelial cell proliferation, as indicated by increased levels of Ki‐67 antigen and proliferating cell nuclear antigen (PCNA). This phenotype was further confirmed in *P. micra*‐infected germ‐free mice, which also exhibited increased levels of both cell proliferation markers. Furthermore, colonic tissues sampled from the infected germ‐free mice displayed increased expression of genes involved in angiogenesis, cell proliferation, invasion, and metastasis. In stark contrast, genes involved in DNA repair and apoptosis were down‐regulated. A soluble factor in *P. micra* conditioned media was also found to induce cell proliferation in HT29 cells in addition to increasing activated β‐catenin and cyclin D1 protein levels together with increased *c‐myc* gene expression. This suggests that an activation of epithelial Wnt/β‐catenin signalling may contribute to CRC progression. In this same mouse gavage model, *P. micra* promoted the expression of many different genes involved in neutrophil, T‐cell, and monocyte chemotaxis in the mouse colons, which is indicative of dysregulated immunity favouring inflammation. Consistent with the observed increased expression of genes encoding Il‐17a, Il‐22, and Il‐23a, colonic infiltration by Th17 cells and IL‐17 staining were both significantly enhanced. To examine this effect more directly, *P. micra* conditioned media were added to CD4^+^ T‐cells isolated from mouse spleens, resulting in their differentiation into an Il‐17^+^CD4^+^ phenotype. These results are consistent with the elevated IL‐17 levels found in the intratumoral environment and the enhancement of tumour growth by IL‐17‐producing CD4^+^ T cells (Wang et al., 2009). The tumour microenvironment can also be influenced by the presence of hydrogen sulfide (H_2_S), a gaseous signalling molecule with diverse functions in the human body (Li et al., 2011). While the exact role of H_2_S in cancer remains to be fully elucidated, it has been recently linked to tumour development (Khattak et al., 2022). Evidence for the oncogenic role of H_2_S can be seen in the overexpression of a number of H_2_S‐producing enzymes in tumours. Tumour‐derived H_2_S stimulates cell proliferation, migration, and angiogenesis (Szabo et al., 2013). Additional sources of H_2_S may also originate from tumour‐associated microbes. In a screen of 37 species of oral bacteria, *P. micra* was found to be the most prolific producer of H_2_S from glutathione (Carlsson et al., 1993). Conspicuously, the tumour‐associated oral pathobiont *F. nucleatum* is also a significant producer of H_2_S (Yoshida et al., 2010). An overabundance of *P. micra*‐derived H_2_S may influence the tumour microenvironment in a number of ways. First, H_2_S is highly toxic at levels above normal signalling functions and exhibits genotoxicity towards intestinal epithelial cells (Attene‐Ramos et al., 2010). Excess H_2_S can exacerbate inflammation (Buret et al., 2022) as well as trigger TCR‐stimulated proliferation of T‐cells, further increasing their inherent capacity to produce H_2_S (Miller et al., 2012). This interplay between both host‐ and bacteria‐derived H_2_S seems like an ideal scenario for the establishment of a positive feedback loop, but it is not yet clear whether such an interaction exists.

## OUTLOOK

The overlapping association of *P. micra*, *F. nucleatum*, and various other prominent oral pathobionts in inflammatory infections and different cancers is both striking and intriguing. For example, periodontal disease has been recently identified as a risk factor for CRC (Janati et al., 2022). It seems somewhat counterintuitive that tumours found in the colon would exhibit infiltration from oral bacteria, given the physical proximity of these tumours to the multitude of colonic bacterial species comprising the most microbially rich environment in the human body. However, sequencing studies of tumour‐associated *F. nucleatum* strains sampled from CRC revealed them to be identical to those found in the oral cavities of the same patients (Komiya et al., 2019). Furthermore, animal model studies have indicated that the origin of *F. nucleatum* transmission to CRC tumours is likely hematogenous, rather than via the GI tract (Abed et al., 2020). Accordingly, many inflammophilic oral pathobionts are also highly tissue invasive. Moreover, bacteremias from the oral cavity occur daily as a consequence of typical behaviours like mastication and routine oral hygiene. For patients experiencing inflammatory oral diseases like periodontitis and odontogenic abscesses, the influx of tissue‐invasive organisms into the bloodstream increases substantially (Forner et al., 2006; Lafaurie et al., 2007). In fact, this phenomenon has been proposed as an explanation for the common observation of oral bacteria in other systemic conditions, such as atherosclerotic plaques (Koren et al., 2011). A similar hematogenous route of infection may be used by *P. micra* as well. It is one of the most highly recovered organisms from the blood following routine dental procedures (Waghmare et al., 2013), while its ability to thrive in serum and establish infections at disparate sites in the body like the liver, brain, and spine further supports hematogenous transmission. The recovery of cultures containing both *P. micra* and *F. nucleatum* in a number of infections involving normally sterile sites (Durovic et al., 2020; Murdoch et al., 1988) demonstrates how certain polymicrobial associations can be remarkably stable. In fact, these interspecies associations may even remain intact during intracellular invasion of epithelial cells, allowing invasive organisms to transport other noninvasive species (Edwards et al., 2006). Thus, it is not difficult to envision analogous scenarios in which motile phagocytes could become stably infected with mixed species of synergistic organisms as well. Currently, it is unclear whether oral bacteria are primarily delivered to extraoral infection sites directly in the blood or indirectly via motile phagocytic immune cells, but this is sure to be an important future line of inquiry. Regardless, there is a wealth of evidence indicating that oral bacteria do survive extensive journeys throughout the human body to establish new infections at distant extraoral sites, which certainly implies the feasibility of oral polymicrobial communities seeding tumour microbiomes.

Despite the plethora of studies indicating the central role of *P. micra* in the pathogenesis of numerous diseases, mechanistic studies of *P. micra* pathobiology are severely lacking. As previously mentioned, only a small number of model organisms from the human microbiome have ever been interrogated at the genetic level, largely due to the paucity of genetic tools available for most microbiome species. This is also true of *P. micra*, as it was long assumed to be a genetically intractable organism. We have a number of ongoing studies attempting to break the genetic barriers currently limiting our access to the inner workings of understudied oral pathobionts. As part of these studies, we recently developed the first robust *P. micra* genetic system, which was demonstrated to function directly in low‐passage clinical isolates. It is now possible to create targeted allelic replacement mutations, perform reporter‐based and tunable gene expression studies, and even employ Tn‐seq compatible forward genetic library screens (Higashi et al., 2022). As such, *P. micra* should now be considered as fully genetically tractable, opening the door for many new exciting lines of inquiry. Understanding how understudied organisms like *P. micra* synergize with other pathobionts, subvert host immunity, and drive dysbiotic disease will undoubtedly yield new paradigms in our future understanding of the human microbiome.

## AUTHOR CONTRIBUTIONS

**Dustin L Higashi:** Conceptualization (equal); methodology (equal); writing – original draft (equal); writing – review and editing (equal). **Madeline C Krieger:** Methodology (equal); writing – original draft (supporting); writing – review and editing (supporting). **Hua Qin:** Writing – original draft (supporting); writing – review and editing (supporting). **Zhengzhong Zou:** Writing – original draft (supporting); writing – review and editing (supporting). **Elizabeth A Palmer:** Writing – original draft (supporting); writing – review and editing (supporting). **Jens Kreth:** Funding acquisition (supporting); writing – original draft (supporting); writing – review and editing (supporting). **Justin Merritt:** Conceptualization (equal); funding acquisition (lead); methodology (equal); supervision (lead); writing – original draft (equal); writing – review and editing (equal).

## CONFLICT OF INTEREST STATEMENT

The authors have no conflicts of interest to declare.

## Data Availability

Data sharing is not applicable to this article as no new datasets were created in this work.
